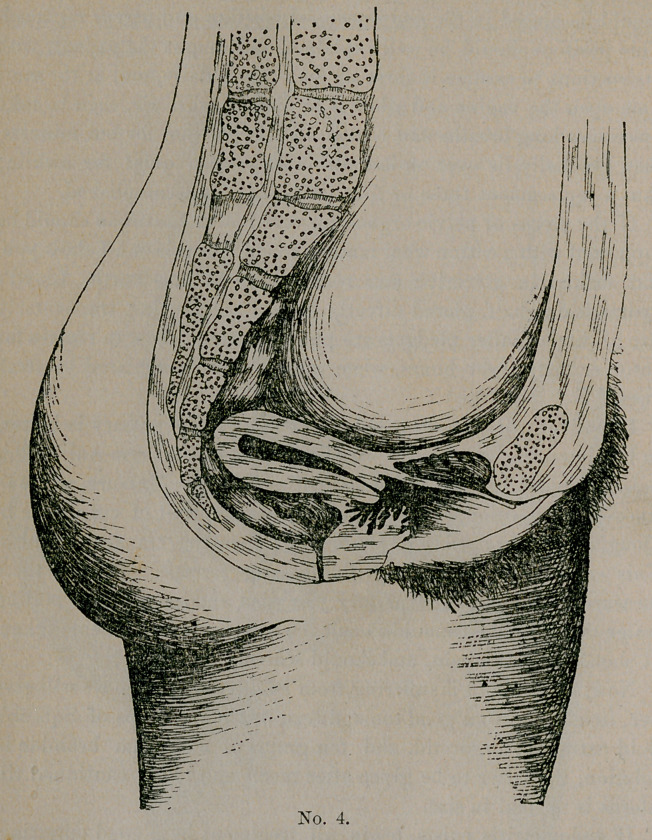# Ante and Retro-Positions or the Uterus—Their Pathology, Symptomatology and Treatment

**Published:** 1893-08

**Authors:** W. W. Stewart

**Affiliations:** Columbus, Ga.


### ANTE AND RETRO-POSITIONS OF THE UTERUS—THEIR PATHOLOGY, SYMPTOMATOLOGY AND TREATMENT.

By W. W. STEWART, M. D.,
COLUMBUS, GA.

To understand fully the malpositions of this organ we must first make ourselves familiar with its normal position, and second, with its supports.

The normal position, as you see in this plate, No. 1 (bladder and rectum empty), represents the normal position both in the virgin and the parous woman. In the virgin it is more anteflected, the normal position, as described by Shultz, when a woman is standing upright and her bladder and rectum empty, being nearly horizontal, more or less anteflected and turned a little to the right.

No. 1.

This position is modified to a certain extent by the repletion of bladder and rectum.

The next question in order is what holds the uterus to its normal axis. This question is plainly answered in plate No. 2. Here we have a diagrammatic outline of all but two of its supports. The two not represented here are the vaginal walls and gravitation with intra-abdominal pressure.

Thus we see the uterus is an organ nicely balanced on elastic cords, well cushioned above by the abdominal viscera and below by the vagina, which also is supportive.

As to the etiology of displacements, I can think of no better classification than that of Thomas and Munde, which I give below as taken from their book.

No. 2.

The general cause of uterine displacements may thus be tabulated:

1. Any influence which increases the weight of the uterus. Influences increasing weight are congestion, tumors in walls or cavity, pregnancy, excessive growth of any of the component parts, subinvolution.

2. Any influence which weakens the uterine support. These are rupture of perineum and posterior vaginal wall, weakening vaginal walls from subinvolution and over distention, stretching of uterine ligaments, relaxation of pelvic fascia, abnormally large pelvis, any influence impairing the sustaining power of the abdominal walls.

3. Any influence which displaces the uterus by pressure, as tight clothing, heavy clothing supported on the abdomen, muscular efforts, abdominal tumors, pelvic inflammatory exudations, repletion of bladder and rectum.

4. Any influence which displaces the uterus by traction; as contracting adhesions following pelvic inflammations, either cellular or intraperitoneal, cicatrices in vaginal walls, shortening of uterine ligament, natural shortness of vagina and uterine ligaments, prolapse of vagina, rectum or bladder.

It is an interesting fact to note that the cervical and cervico-corporal flexions are most frequent in the nulliparous woman, while the corporal form is most often found in the multiparous.

Retroflections are most usually found in cases in which there has been a weakening of the tone of the uterine wall, although it is often caused by direct force, whether of rapid or slow development. The uterus may be either anteflexed, retroflexed or lateroflexed, or ante-lateroflexed, or retro-lateroflexed. All of these varieties have various complications, the most prominent of which is ascent and descent.

There are still two other forms, ante-position with retroflexion and retro-position with anteflexion.

Symptoms: Pain in back and loins, weighty, dragging sensation in the pelvis. Constipation in retro-position—vesical and rectal tenesmus in both. Great fatigue from walking, much complained of below the knees, lassitude and inability to lift weights, leucorrhœa and other signs of congestion.

In addition to these I must mention a uterine syndrome, which is quite interesting and at the same time of great aid often in obscure cases where sufficient direct symptoms are not complained of to suggest to one not accustomed to handle many of these cases the advisability of an examination.

1. Pain of a bearing down nature in the top of the head, caused by endometritis or vesical irritation.

2. Pseudo-angina with palpitation of gastric origin.

3. Irritative or indolent digestion with dilatation of the stomach. I would beg of you to remember this fact, for it will, I am sure, prove not only of interest but of profit to all. This dyspepsia is caused by the endometritis associated with flexion. The explanation of this is found in the peculiar richness of the sympathetic innervation of both the uterus and stomach, and is of reflex origin. Its line of symptoms are nausea, loss of appetite, vomiting directly after eating, it being rather regurgitant in character; flatulency, which occurs in the form of a chronic tympanites, the patient's stomach continuing to enlarge though she may have lost flesh.

4. A dry, hacking cough, which is paroxysmal and comes usually in three or five coughing efforts at a time. So troublesome is this that the patient who suffers therefrom cannot sleep at times. No inflammation of chest, pharynx or larynx can be detected, and it disappears with the uterine trouble.

5. Pain in either breast, usually the left.

6. Pain of a dull character in the wrists, inner side of the palms, and last phalanges.

7. Hysterical phenomena whose name is legion.

8. The fascia uterina, which you will recognize in a habitual chloro-anemic color, muddy complexion, dark circles under the eyes, together with a pinched, suffering face, which completes the picture. In addition to these there are, of course, many symptoms peculiar to each individual case.

To illustrate this fully I will mention a case of mine which bears upon this point: Miss———, age thirty; occupation dressmaker, came under my care for dyspepsia and vesical tenesmus. For five years had been a great sufferer with pelvic pains, vesical and rectal tenesmus. Headaches, occipito-parietal and parietal in location; constipation at times, at others long continued attacks of diarrhœa, spitting up of food directly after eating, tympanites so marked that she was ashamed to go on the street. Had lost flesh for months prior to coming under my care. Measurement of stomach at umbilicus twenty-nine; three inches below thirty-five inches. Muscular walls tense. Leucorrhœa was quite bad, causing great discomfort and decided excoriation. Urine alkaline, no albumin or sugar. Stomach would easily hold six glasses of water without discomfort, when loud succussion sounds could be elicited. Percussion showed the stomach greatly dilated. Abdomen generally hyperæsthetic. Uterus anteflexed with decided endometritis. Patient was taken off of all starchy and saccharine food and given proper tonics and uterine trouble properly treated. When discharged had gained fourteen pounds, and stomach measurements were as follows: At umbilicus twenty-two; three inches below twenty-seven inches. Stomach was still slightly dilated, but caused no dyspeptic symptoms. All other symptoms disappeared.

PATHOLOGY.

Flexions and versions, either anterior or posterior, are often congenital by an excessive nutrition in the anterior or posterior wall, as the case may be; the excessive growth causing a convexity in the side most highly nourished, and a concavity in the opposite.

Rokitanskey has proven that in a perfectly developed uterus weakening in its wall is often caused by endometritis, which creates an inward growth of the utricular glands into the submucosa near the os internum, which in consequence undergoes atrophy and enfeeblement.

Klobe gives as a frequent cause cystic degeneration of the cervical glands, which, from their increased size and subsequent pressure, bursting thereby, cause a collapse of tissue in the formerly dense framework of the uterus, leaving in its place a flaccid net-like areolar tissue incapable of sustaining the uterus in its normal position.

The uterus being once flexed, either in the anterior or posterior position, there at once arises an acute congestion followed by sub-acute and chronic in the majority of cases. Especially is this true in cases of sudden displacement, and it holds good to a modified degree in all cases.

This is caused by veins with their flaccid walls being closed at the point of flexion, while the arterial walls, being rigid, remain open and convey blood into an organ already full, thus causing the acute congestion. The flexion cuts off the return flow through the hypogastrics, thereby throwing all the work upon the inadequate spermatic veins. As a result of this the pampiniform flexus is constantly overdistended, and an œdematous boggy condition is set up in ovaries, tubes, broad ligaments and uterus, making the flexion worse, at the same time setting up a tendency to hydrosalpinx and cystic degeneration and formation in ovaries and broad ligaments.

One of the most prominent pathological lesions with which we have to deal as causative in displacements is slight infection at parturition, causing subinvolution with metritis, parametritis, followed by contraction of uterine ligaments.

After thus hurriedly considering the etiology, pathology and symptomatology of these troubles, we will now take up the cases themselves and discuss them, endeavoring at the same time to bring out the treatment in each case.

Here in plate No. 3 we have a simple retroversion with beginning prolapse caused by subinvolution and relaxation of the uterine ligaments, vaginal walls and perineal fasciæ. This is a case in which, if of recent origin, we can look for good and quick results. Having made first a clear diagnosis of the cause (and I would state here that is the keynote to the successful treatment), we will begin our treatment, which, in all displacements, will be along certain general lines. These we will give now and not repeat:

1. Remove all weight from the hips and constrictions from the waist by using skirt supporters.

2. Measure your patient at the line of the umbilicus, or just above, and secure for her a corset waist two inches smaller than waist measure.

3. If abdominal walls are pendulous and relaxed use an abdominal belt made for the patient in question.

4. Require a regular amount of daily exercise, to be gradually increased, that will not overtax the strength.

5. Order alternative hot and cold douches to be taken in the dorsal position. Use the fountain syringe only, with one quart in quantity—patient to remain in recumbent position for thirty minutes after taking.

No. 3.

The alternate hot and cold douche tends to cause contraction of the muscular fibers of the uterus and uterine ligaments, thus bringing about good nutrition and muscular tone, as is produced by electricity. The cold douches should be taken last.

6. Guard well and build up your patient's general condition.

As to the special treatment of this case in hand, the patient should be placed in the genu-pectoral position and uterus replaced. This position should be well explained and taught the patient, with instructions to assume it three or four times daily, and at the same time open the vagina and allow it to distend with air, then to take successive long breaths and remain in this position for ten minutes, then gradually lie over on her side and remain quiet for awhile; thus a replacement three or four times daily is accomplished.

If the uterus or pelvic organs are very tender the organ should be supported with a cotton wool tampon made like this one I show you, and soaked in glycerine and rose water āā one-fourth, Lloyd's hydrastis one-half, placed directly behind the cervix; transversely two or three smaller pledgets are placed in front; these to remain for not over forty-eight hours, when they should be replaced by fresh ones.

If patient can tolerate it a hard rubber Hodge pessary is better, as it allows the douches to be taken without its removal. This pessary should be *fitted* to the patient as follows: Measure, while in knee-chest position, distance from posterior wall of cul-de-sac of Douglas to inner surface of pubic arch; then the transverse width. This can be nicely done with dressing forceps. Having these measurements, select the pessary you wish and emerse it in boiling water till soft; then mould to suit the case by measurements taken. It should not give pain, and should scarcely be felt.

Now, this patient is suffering from subinvolution; so we will give her, in addition to a good tonic, fifteen or twenty drops of Squibb's fluid extract of ergot tid. and ten grains of potassium bromide in solution, the latter to be given after meals and to be continued till uterus is normal in size.

If endometritis exists, begin all treatment by a good curetting, and pack uterus with a long strip of iodoform gauze, to be removed on the third day.

The laws of antisepsis should be carefully carried out throughout the treatment.

Now in this plate, No. 4, we have a very interesting case, bringing out strongly another frequent cause for displacement.

No. 4.

Miss G——, aged twenty-five, when twelve years old fell upon a steelyard hook, the hook entering the vagina and tearing the tissues badly, its course being upward and outward against the inner surface of the pubic bone.

Had been a sufferer in the long line of symptoms so familiar to us all, till she fell into my hands five months ago. On examination, the condition you see here represented was found. The vesico-uterine ligaments were taut; cervix fixed in its anterior and prolapsed position; uterus retroverted.

Patient placed in genu-pectoral position and uterus returned as far as practicable to its normal position. Then I began to stretch the vesico-uterine ligaments by daily pushing the cervix backward as far as she could tolerate, and after ten days of this treatment succeeded in getting it back sufficiently to use a figure-of-8-pessary, which she wore with comfort for one month, when length was increased till uterus was in normal position and freely movable.

The uterus was decidedly sinistro-rotated, and when pessary was removed would immediately return to its former position to a certain extent.

Thinking it was one of those cases in which Alexandre's operation would be of benefit, I operated and found that the hook, which entered the vagina on the right side, had torn out the round ligament, causing the sinistro displacement; so was compelled to sew up the wound and depend upon a figure-of-8-pessary to do the work.

*(Continued.)*

## Figures and Tables

**No. 1. f1:**
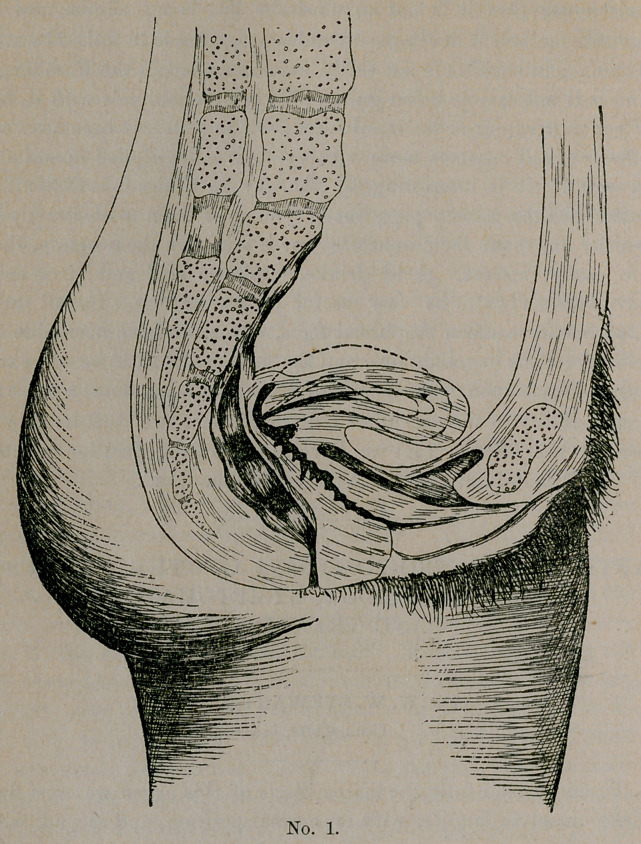


**No. 2. f2:**
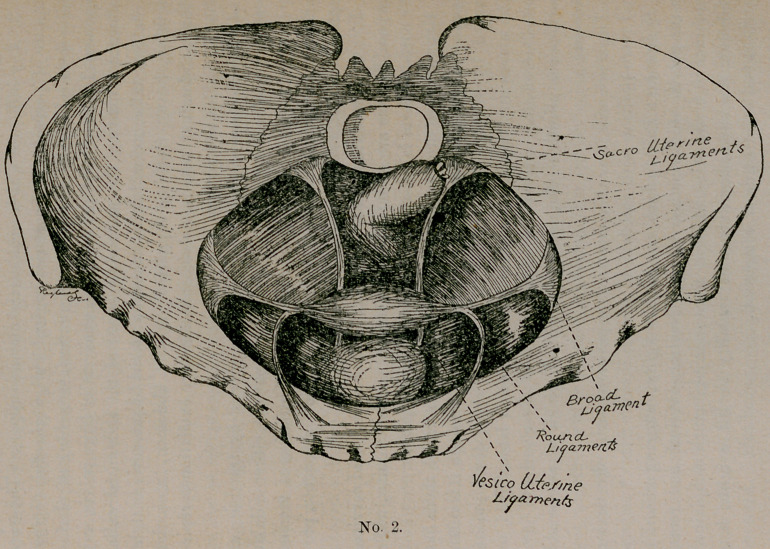


**No. 3. f3:**
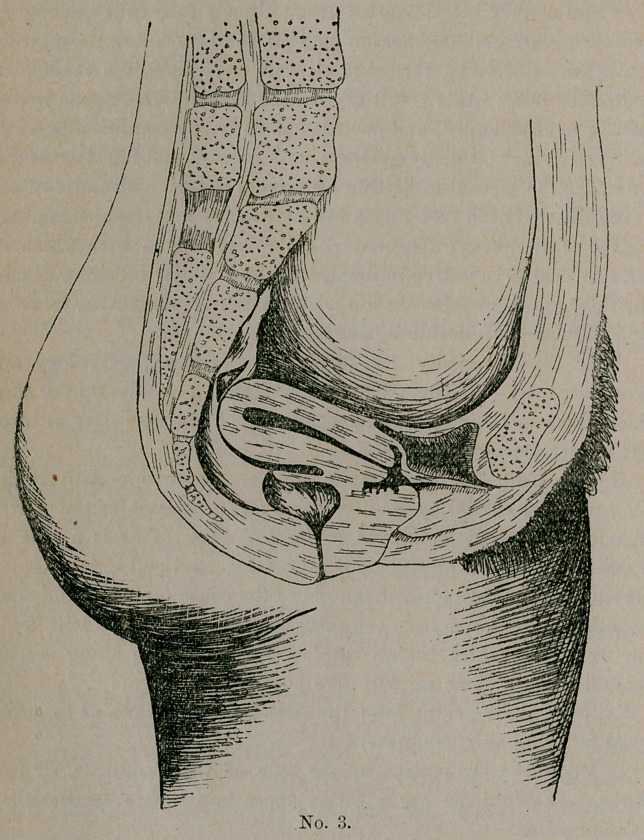


**No. 4. f4:**